# Relationship Between Vitamin D Deficiency and Postpartum Depression

**DOI:** 10.3390/jpm15070290

**Published:** 2025-07-04

**Authors:** Ioanna Apostolidou, Marios Baloukas, Ioannis Tsamesidis

**Affiliations:** 1 Department of Midwifery, International Hellenic University, 57001 Thessaloniki, Greece; itsamesidis@auth.gr; 2 Department of Physiotherapy, International Hellenic University, 57001 Thessaloniki, Greece; phy58221@phys.ihu.gr; 3 Department of Biomedical Sciences, International Hellenic University, 57001 Thessaloniki, Greece

**Keywords:** vitamin D, postpartum depression, maternal mental health, pregnancy, hypovitaminosis D, antenatal depression, nutritional deficiency, perinatal care, 25-hydroxyvitamin D, mental health

## Abstract

**Background/Objectives:** Postpartum depression (PPD) affects approximately 10–20% of women during and after pregnancy, posing significant risks to maternal health, infant development, and family dynamics. Identifying modifiable risk factors is essential for prevention. Emerging evidence suggests that vitamin D, a neuroactive steroid hormone involved in neurotransmitter synthesis, neuroinflammation regulation, and calcium homeostasis, may play a protective role against mood disorders, including PPD. **Methods**: The search was conducted through a comprehensive search of the PubMed, Scopus, and Web of Science databases using a combination of Medical Subject Headings (MeSH) and free-text terms including “vitamin D”, “25-hydroxyvitamin D”, “deficiency”, “pregnancy”, “postpartum”, “depression”, “antenatal depression”, “maternal mental health”, and “perinatal mood disorders”. **Results**: Numerous observational studies and systematic review reports around the world reinforce the potential global relevance of vitamin D insufficiency. This study advances personalized and precision medicine approaches by emphasizing the importance of individualized screening for vitamin D deficiency during pregnancy and postpartum, enabling tailored interventions that could mitigate the risk of postpartum depression. **Conclusions**: In conclusion, while a definitive causal relationship between vitamin D deficiency and perinatal depression remains unproven, screening for vitamin D levels during pregnancy could serve as a low-risk intervention to support maternal mental health. Future research should focus on well designed, large-scale randomized trials and standardization of diagnostic criteria to clarify vitamin D’s role in preventing perinatal depression. Recognizing vitamin D status as a modifiable biomarker allows for targeted nutritional and pharmacological strategies to optimize maternal mental health.

## 1. Introduction

Postpartum depression (PPD) is a prevalent and often underdiagnosed mental health condition that significantly impacts maternal well-being and child development. Affecting approximately 10–20% of women globally, it can have long-term consequences for both mother and infant if left untreated [1]. Among the various biological, psychological, and social factors implicated in the pathophysiology of PPD, increasing attention is being directed toward the potential role of vitamin D deficiency during pregnancy and the postpartum period [2]. The biological mechanisms involve vitamin D’s role in brain function, immune response regulation, and neurotransmitter synthesis. There is growing evidence linking vitamin D deficiency to depression, including postpartum depression (PPD). The biological plausibility hinges on vitamin D’s roles in neuroinflammation regulation, neurotransmitter synthesis, and neuroplasticity [3]. Multiple studies suggest that low vitamin D levels may increase the risk of depression during and after pregnancy, though the findings are inconsistent and are affected by study design differences [4]. The biological plausibility involves vitamin D’s role in brain development, neurotransmitter function, and immune regulation. While some studies show a clear association, others have inconsistent results, possibly due to differing methodologies and the timing of vitamin D assessment [5]. Some studies show biological evidence that supports vitamin D’s neuroprotective and anti-inflammatory functions, which could influence mood regulation during and after pregnancy [6]. Nutritional deficiencies, especially vitamin D and omega-3 fatty acids, are consistently associated with an increased PPD risk. Nutritional factors, particularly vitamin D, are critical modulators of postpartum depression (PPD). A substantial body of observational evidence links nutritional deficiencies (e.g., vitamin D, folate, and iron) to an increased PPD risk, emphasizing biologic pathways involving neuroinflammation and neurochemical imbalances [7]. Vitamin D, a fat-soluble vitamin primarily synthesized through the exposure of the skin to sunlight, plays a pivotal role in calcium homeostasis and immune function. Emerging evidence also suggests its involvement in neuropsychiatric regulation, particularly in the synthesis of neurotransmitters (serotonin) and the modulation of inflammatory pathways [8]. Given the immunomodulatory and neuroprotective properties of vitamin D, its deficiency may contribute to the onset or exacerbation of depressive symptoms in vulnerable populations such as pregnant and postpartum women. Significance of the Topic: Vitamin D deficiency is highly prevalent in pregnant women due to altered metabolism, limited sun exposure, and dietary insufficiencies, raising concerns about its impact on maternal mental health [9]. Several observational studies and meta-analyses have reported associations between low serum vitamin D levels and an increased risk of antenatal and postpartum depressive symptoms [4,10]. However, conflicting findings in other studies necessitate a comprehensive synthesis of the available literature to evaluate the consistency and strength of this association. Research Gap: While multiple systematic reviews and individual studies have addressed the relationship between vitamin D status and PPD, heterogeneity in the study designs, population demographics, assessment tools, and vitamin D measurement methods limits the generalizability of findings. Moreover, most available reviews have not holistically integrated both gestational and postpartum vitamin D exposure in relation to maternal depressive symptoms. PPD is a multifactorial condition influenced by genetic, hormonal, psychological, and social factors. Recently, vitamin D deficiency (VDD) has gained attention as a potential contributor due to vitamin D’s extensive biological roles beyond calcium regulation. Vitamin D receptors (VDRs) are widely distributed in the brain, including the hippocampus, hypothalamus, and prefrontal cortex, which are areas that are intimately involved in mood regulation. The active form of vitamin D, 1,25-dihydroxyvitamin D, also contributes to serotonin synthesis, neuroimmunomodulation, and calcium homeostasis in neuronal cells, all of which are implicated in the pathophysiology of depression [11]. Growing evidence suggests a link between VDD and an increased risk of depressive symptoms during the perinatal period. Several observational and clinical studies, summarized in systematic reviews, have found that low serum 25(OH)D levels during pregnancy are significantly associated with a higher risk of PPD. For example, Robinson et al. (Australia) reported that women in the lowest quartile of vitamin D levels had a significantly increased risk of PPD symptoms, while Fu et al. (China) found that higher serum vitamin D levels correlated with reduced PPD symptoms. Additionally, a 2020 systematic review concluded that six out of eight studies demonstrated a positive association between VDD and both gestational and postpartum depression, suggesting vitamin D supplementation may be a promising strategy for reducing depressive symptoms [11].

Nevertheless, not all of the findings are consistent. Some large-scale cohort studies have reported no significant association or even a U-shaped relationship, where both low and high serum vitamin D levels were linked with an elevated PPD risk [4]. These discrepancies may stem from differences in the study designs, timing of vitamin D measurement, population demographics, diagnostic criteria, and the presence of confounding factors such as seasonality, supplementation, and inflammatory status [4,12].

Vitamin D deficiency and postpartum depression (PPD) are both significant global public health concerns [13]. Postpartum depression affects a considerable proportion of new mothers with wide-ranging consequences for both maternal and infant health [11]. Research suggests vitamin D may play a protective role in mood regulation. Studies, including those among Taiwanese, Iranian, Turkish, and other populations, suggest that low vitamin D levels are associated with higher risks of developing PPD [11]. Vitamin D’s role as a neuroactive steroid that is essential for brain development and neurotransmitter regulation underlies its potential involvement in mood regulation. PPD affects approximately 10–20% of women globally [3,11].

From a personalized medicine perspective, understanding the individual variability in vitamin D metabolism and its impact on mental health is crucial. Genetic factors, lifestyle, and environmental exposures can all influence vitamin D status and its subsequent effects on mood regulation. Therefore, identifying women at risk of PPD through a personalized risk assessment, including vitamin D screening, could lead to targeted interventions and improved outcomes [11,14].

## 2. Objective

This narrative review aims to synthesize findings from recent observational and interventional studies on the association between vitamin D deficiency and postpartum depression. Emphasis is placed on both serum 25(OH)D levels and vitamin D supplementation during pregnancy. By evaluating the strength, consistency, and biological plausibility of this relationship, this review seeks to determine whether vitamin D might serve as a modifiable risk factor for postpartum depression [15].

## 3. Materials and Methods

This narrative review was conducted to explore the relationship between vitamin D status and the incidence of depression during the gestational and postpartum periods. A structured yet flexible approach was adopted in accordance with general guidance for narrative reviews, prioritizing comprehensiveness and relevance over exhaustive inclusion. The process involved the identification, selection, and critical analysis of both observational and interventional studies published in peer-reviewed journals.

### 3.1. Literature Search Strategy

Relevant articles were identified through a comprehensive search of the PubMed, Scopus, and Web of Science databases. The search was conducted using a combination of Medical Subject Headings (MeSH) and free-text terms including “vitamin D”, “25-hydroxyvitamin D”, “deficiency”, “pregnancy”, “postpartum”, “depression”, “antenatal depression”, “maternal mental health”, and “perinatal mood disorders”. Boolean operators (AND, OR) were used to refine the searches. The search was restricted to English-language articles published between 2000 and 2024 to capture both foundational studies and recent developments in the field. The reference lists of the retrieved articles were also manually screened to identify additional relevant studies not captured in the database search. The PRISMA 2020 checklist (Supplementary Table S1) confirms adherence to reporting standards.

### 3.2. Inclusion and Exclusion Criteria

A priori eligibility criteria were defined to focus the review on relevant, high-quality evidence and to minimize selection bias. Studies were included if they were published in peer-reviewed journals and explicitly examined the relationship between maternal vitamin D status (assessed via dietary intake, serum 25(OH)D concentrations, or supplementation) and depressive symptoms or clinical diagnoses during pregnancy or the postpartum period. Eligible study designs comprised observational studies (cross-sectional, case–control, or cohort) and interventional trials (specifically randomized controlled trials) conducted in populations of pregnant or postpartum women. Studies were excluded if they were not available in English, if they focused exclusively on non-perinatal populations (e.g., women or men outside the pregnancy/postpartum context), or if they lacked original empirical data (such as commentaries, editorials, narrative reviews, or other opinion pieces). Each criterion was chosen to maximize the relevance and methodological rigor of the included literature and to ensure transparency.

Published in peer-reviewed journals: Ensures inclusion of rigorously vetted studies. Peer review serves as a basic quality filter in evidence synthesis; narrative literature reviews typically rely on peer-reviewed original research. This criterion helps exclude unverified sources (e.g., unpublished or low-quality reports) and aligns with standard practices for reviews.

Vitamin D–depression association during pregnancy/postpartum: Focuses on the specific exposure and outcome of interest. By requiring each study to directly examine maternal vitamin D status and antenatal/postpartum depressive symptoms or diagnoses, the criterion guarantees relevance to the research question and avoids unrelated work.

Observational (cohort, case–control, cross-sectional) or interventional (RCT) designs: Captures the primary study types that can address exposure–outcome associations and causal effects. Observational designs are well-suited to investigate associations between an exposure and a health outcome, and they provide real-world epidemiological evidence. Randomized controlled trials are the gold standard for assessing causal effects of vitamin D interventions. Including both design categories ensures a comprehensive evidence base.

English language: Facilitates accurate interpretation of study methods and results. Limiting to studies reported in English is a pragmatic choice to ensure the reviewers can reliably assess eligibility and extract data. This is a common practice in reviews and is noted as a reason for exclusion when language resources are constrained.

### 3.3. Exclusion Criteria

1. Were not available in English.
2. Focused solely on populations outside the perinatal period.
3. Were commentaries, editorials, or non-systematic opinion pieces.

Study Selection and Data Synthesis: A total of 26 studies were selected based on their relevance and methodological quality. These included systematic reviews [11], randomized controlled trials, and observational studies [14].

The selected literature provided data on vitamin D measurement methods, population characteristics, study settings, and depression assessment tools (e.g., EPDS, BDI, and DSM criteria). Key findings were synthesized thematically rather than quantitatively, with an emphasis on identifying consistencies and divergences across studies. Particular attention was given to study design, timing of vitamin D assessment, and diagnostic criteria for depressive symptoms.

Each criterion was justified by methodological and clinical rationale. For example, peer-review and specified designs enhance evidence quality (consistent with reporting standards), and the population restriction preserves applicability to maternal mental health. Transparent reporting of these predefined criteria—with explicit rationale as above—is aligned with PRISMA and related guidelines for clear, reproducible literature searches.

### 3.4. Data Organization Strategy

The data from the selected studies were organized through a thematic synthesis approach, which is typical of narrative reviews. Studies were grouped based on the nature of their contribution (e.g., epidemiological evidence, biological mechanisms, and interventional outcomes), and findings were qualitatively analyzed to identify common patterns, divergences, and underlying mechanisms. Tables and comparative analyses were constructed to illustrate consistencies and contextual differences across study populations and methodologies. This organization allowed for a comprehensive interpretation of the current literature on vitamin D deficiency and postpartum depression without attempting a meta-analytic quantification.

### 3.5. Validation Strategy

Although a formal quality appraisal tool was not employed, dual independent screening and cross-verification of extracted data were used to improve reliability. Calibration discussions were held during initial stages to ensure consistent application of inclusion criteria and thematic coding.

### 3.6. Ethical Considerations

As this was a review of published literature, no ethical approval was required.

### 3.7. Review Protocol and Registration

No formal review protocol was registered in PROSPERO or any other public database, as this study was conducted as a narrative review rather than a systematic review. While narrative reviews do not typically require formal registration, the authors followed a structured and transparent methodology in accordance with best practices for narrative synthesis, including defined inclusion criteria, thematic organization of findings, and dual reviewer screening.

## 4. Results—Epidemiological Evidence of Vitamin D Deficiency and Postpartum Depression

### 4.1. Prevalence and Distribution of Vitamin D Deficiency

Vitamin D deficiency is a global phenomenon affecting diverse populations. One systematic review indicated that over one billion individuals worldwide suffer from vitamin D deficiency, with prevalence rates ranging from 30% to 90% in different regions [16]. Immigrant populations in Europe, for example, often exhibit lower serum 25-hydroxyvitamin D (25(OH)D) concentrations compared to indigenous populations due to factors such as a darker skin pigmentation and lifestyle practices [17]. A study carried out in Taiwan found that women who observed traditional confinement practices during the postpartum period exhibited significantly lower plasma levels of riboflavin, which was correlated with an increased risk of PPD [18]. Similarly, research conducted among Iranian women demonstrated that those with postpartum depression had significantly lower levels of vitamin D (mean: 16.89 ng/mL) compared to nondepressed controls (mean: 21.28 ng/mL) [5]. Notably, women with vitamin D levels below 20 ng/mL were found to be over three times more likely to develop PPD (Odds Ratio: 3.3) [5].

### 4.2. Prevalence of Postpartum Depression

The prevalence of postpartum depression is widely variable. In the Taiwanese study, the prevalence was reported at 8.4% [18], while systematic reviews from other regions have identified PPD prevalence rates ranging between 18% and 19% [11]. The diversity in prevalence rates can be partially attributed to differences in diagnostic criteria, screening tools, and cultural practices.

Additionally, confounding factors such as nutritional status and psychological stress have been observed to impact the risk profile for PPD [18].

Table 1 summarizes selected studies that document the relationship between vitamin D deficiency and postpartum depression.

These findings underscore that vitamin D deficiency is not only widespread across diverse demographic groups but is also significantly associated with the onset of postpartum depression in different cultural contexts. While epidemiological studies indicate an association between vitamin D deficiency and postpartum depression, understanding the biological basis of this link remains crucial for developing targeted interventions. Exploring the underlying mechanisms can shed light on how VDD may influence PPD risk.

### 4.3. Biological Mechanisms Linking Vitamin D Deficiency and Postpartum Depression

Recent research has increasingly focused on the biological plausibility underlying the link between vitamin D deficiency and postpartum depression. Vitamin D functions as a neuroactive steroid: it modulates neurotransmitter synthesis, influences neurotrophic factor production, and regulates calcium homeostasis—all of which are crucial for maintaining optimal brain function [12,14]. Vitamin D exerts its effects in the central nervous system through multiple inter-related mechanisms involving neuroendocrine, immune, and neurotransmitter pathways. The biologically active form of vitamin D, 1, 25-dihydroxyvitamin D (calcitriol), binds to vitamin D receptors (VDRs) that are abundantly expressed in key brain regions such as the hippocampus, amygdala, prefrontal cortex, and hypothalamus, all of which are critical in mood regulation. 

### 4.4. Neurotransmitter Modulation

Vitamin D plays a key role in the biosynthesis of serotonin, acting via transcriptional regulation of the tryptophan hydroxylase 2 (TPH2) gene. This enzyme is essential for the conversion of tryptophan to serotonin in the brain. Low levels of vitamin D result in impaired serotonin production, which is associated with mood disorders, including PPD. In addition, calcitriol modulates dopaminergic and noradrenergic activity, which are both implicated in affective disorders [13,15,19].

### 4.5. Neuroinflammation and Cytokine Regulation

Vitamin D has notable anti-inflammatory properties, downregulating pro-inflammatory cytokines such as IL-6, TNF-α, and IL-1β, and upregulating anti -inflammatory markers such as IL-10. Since elevated inflammatory cytokines have been strongly associated with perinatal depression, particularly postpartum, vitamin D may counteract neuroinflammatory pathways involved in depressive symptomatology [15,16,20].

### 4.6. Neurotransmitter Regulation and Neuroprotection

Vitamin D receptors (VDRs) are widely expressed in brain tissues, which is central to its role as a neuroactive steroid. The biologically active form of vitamin D, calcitriol, has been shown to influence neurotransmitter pathways by modulating the synthesis and release of serotonin and dopamine [16,21]. Abnormalities in these neurotransmitters have been implicated in depressive disorders. Moreover, vitamin D’s neuroprotective properties may counteract the deleterious effects of neuroinflammation and oxidative stress, which is a proposed mechanism in the pathophysiology of postpartum depression [3,16].

### 4.7. Inflammatory Mediators and HPA Axis Modulation

Several studies have highlighted that vitamin D plays a critical role in down regulating inflammatory cytokines. Increased inflammation is a common finding in depression, and higher levels of inflammatory markers have been positively correlated with depressive symptomatology [18]. In postpartum women, the concomitant presence of vitamin D deficiency and an overactive inflammatory response may potentiate the dysfunction of the hypothalamic–pituitary–adrenal (HPA) axis—a key factor in mood disorders [11,18]. Disruption of the hypothalamic–pituitary–adrenal (HPA) axis is a well-established feature in mood disorders. Vitamin D is involved in regulating the expression of glucocorticoid receptors and reducing circulating levels of cortisol, potentially stabilizing stress reactivity. In postpartum women, low vitamin D may exacerbate HPA axis dysregulation, contributing to emotional instability and depressive symptoms.

### 4.8. Epigenetic and Microbiome-Related Pathways

Recent findings suggest that vitamin D can influence epigenetic regulation, including DNA methylation of genes involved in stress and mood regulation. Moreover, vitamin D modulates the gut–brain axis by shaping the intestinal microbiota, which in turn can affect systemic inflammation and mental health—a promising new area of exploration in PPD research.

**Figure 1:**
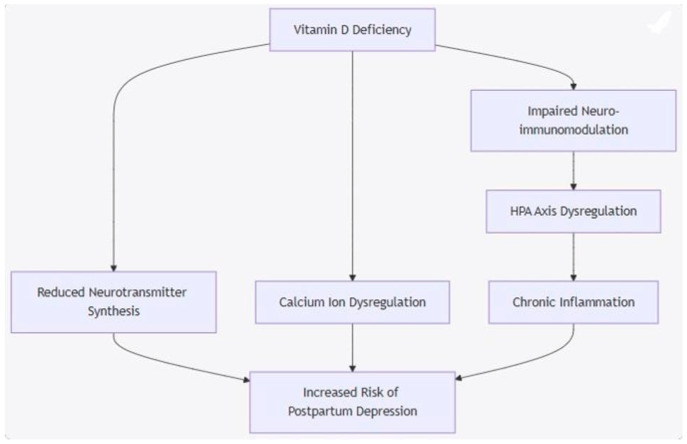
Proposed Biological Mechanism Diagram. Mermaid flowchart diagram that summarizes key biological pathways by which vitamin D deficiency may lead to postpartum depression.

While these mechanistic insights provide plausible explanations, clinical studies are essential to determine whether vitamin D supplementation can successfully reduce the risk or severity of postpartum depression. Also, while understanding the biological pathways is crucial, determining how these mechanisms translate to clinical outcomes requires further investigation through human studies (Figure 1).

### 4.9. Clinical Studies and Comparative Analysis

A number of clinical studies have evaluated the relationship between vitamin D levels and postpartum depression, employing various methodological approaches. This section summarizes key findings from the literature and explores both the convergent and divergent results.

#### 4.9.1. Taiwanese Population Studies

One study conducted among Taiwanese women, who traditionally practice postpartum home confinement, reported a significant association between nutritional status—particularly plasma riboflavin levels—and postpartum depression [18]. Women with PPD exhibited riboflavin levels that were 13.9% lower compared to their non-depressed counterparts.

Additionally, traditional confinement practices further predisposed these women to greater psychological stress and lower satisfaction with postpartum care [5,18].

These findings suggest that cultural practices and nutritional deficiencies may have a synergistic effect on the development of depressive symptoms during the postpartum period [18].

#### 4.9.2. Iranian and Other International Studies

Research among reproductive-aged Iranian women revealed that those experiencing postpartum depression had significantly lower concentrations of vitamin D compared to healthy controls [5]. The study reported that 53.3% of depressed women had vitamin D levels below 20 ng/mL, a percentage that was markedly higher than that found in the control group (31.7%). The calculated odds ratio of 3.3 indicated a robust association between vitamin D deficiency and the risk of developing postpartum depression [5].

Another dimension is provided by studies in immigrant populations in Europe, where vitamin D deficiency was compounded by limited sunlight exposure and cultural clothing practices.

These studies demonstrated that nonwestern immigrant groups had substantially lower 25(OH)D levels compared to indigenous Europeans, highlighting important environmental and genetic determinants of vitamin D status that may indirectly influence mood disorders [17].

#### 4.9.3. Systematic Review Findings

A systematic review evaluating the associations between vitamin D status and depressive symptoms during and after pregnancy compiled data from multiple studies with varying outcomes [11].

The review found that 55% of studies on postpartum depression and 71% of studies on antenatal depression showed a significant association with vitamin D levels. However, due to the heterogeneity in study designs, diagnostic thresholds for vitamin D deficiency, and outcome measures, a meta-analytic synthesis was not feasible [11].

#### 4.9.4. Comparative Analysis Table

The table below provides a comparative synthesis of key clinical studies assessing the relationship between vitamin D deficiency and postpartum depression (Table 2).

This comparative analysis highlights that despite variations in study designs and population characteristics, a common thread is the association between reduced vitamin D levels and an increased risk of postpartum depression (Table 2).

### 4.10. Intervention Studies and Recommended Vitamin D Intake

Evidence from intervention studies suggests that vitamin D supplementation may have potential benefits in improving mood and reducing the risk of postpartum depression. For instance, recommendations from various nutritional guidelines suggest that an intake of ≥800 IU/day of vitamin D can be effective in reducing fracture risk in older subjects and may also support mental health in postpartum women [20]. Although most studies have focused on the benefits of vitamin D in bone health, emerging evidence indicates that similar supplementation regimens could be beneficial for mood regulation in depressive disorders [16,19].

Clinical trials have explored loading protocols to quickly reach optimal 25(OH)D levels followed by maintenance doses. One approach discussed in the literature involved a loading regimen to treat vitamin D deficiency in adults, ensuring that the target serum 25(OH)D concentration is rapidly attained before transitioning to a stable maintenance dose. While data specifically linking such protocols to improvements in PPD are still emerging, the rationale for vitamin D supplementation is supported by both epidemiological correlations and biological plausibility [19,20].

Despite promising findings, the existing body of clinical research faces several challenges that must be addressed to draw definitive conclusions.

### 4.11. Limitations, Future Directions, and Implications for Public Health

#### 4.11.1. Limitations in Current Research

Despite the growing body of evidence linking vitamin D deficiency with postpartum depression, several limitations remain:

Heterogeneity in Study Designs and Diagnostic Criteria: Variation in the diagnostic thresholds for vitamin D deficiency (e.g., >50 nmol/L versus >75 nmol/L) and differing depression assessment tools (EPDS, Beck Depression Scale) complicate direct comparisons between studies [11]. This heterogeneity hinders the integration of findings into a coherent meta-analytic framework [11].

Confounding Factors: Many studies have not fully adjusted for potential confounders such as concurrent nutritional deficiencies (e.g., riboflavin and n-3 PUFA), socioeconomic status, lifestyle factors, and genetic predispositions (e.g., VDR gene polymorphisms). Without controlling for these variables, the direct contribution of vitamin D deficiency to the risk of PPD remains difficult to isolate [18].

Methodological Variability in Vitamin D Measurement: The assays used to measure serum 25(OH)D levels—including ELISA, chemiluminescence assays, and LC-MS/MS–vary in their sensitivity and specificity, contributing to variability across studies [17,20]. This methodological inconsistency can lead to discrepancies in defining vitamin D status and its association with depressive symptoms [20].

Cultural and Environmental Influences: Cultural practices, such as traditional postpartum confinement in Taiwanese women, and environmental factors, such as sunlight exposure, add layers of complexity to the interpretation of results [17,18]. These factors are often region-specific, thereby limiting the generalizability of study findings to broader populations [17].

To overcome these limitations, future studies should aim for more standardized methodologies and randomized controlled trials to establish causality and effective interventions.

#### 4.11.2. Future Research Directions

Given these limitations, future research should have the following aims: 

Standardize Methodologies: Future studies should adopt uniform diagnostic criteria for vitamin D deficiency and use standardized assays to measure serum 25(OH)D levels. This would facilitate easier pooling of data and more reliable comparisons across diverse populations. Incorporate Comprehensive Nutritional Assessments: Studies should evaluate vitamin D status alongside other critical micronutrients (e.g., B vitamins) to better understand the combined effect of nutritional deficiencies on PPD risk. Multivariate models that account for dietary intake, sunlight exposure, and other lifestyle factors would provide more robust conclusions. Conduct Longitudinal and Interventional Studies: Longitudinal studies can help ascertain the temporal relationship between vitamin D deficiency during pregnancy and the subsequent development of postpartum depression. Additionally, randomized controlled trials of vitamin D supplementation in at-risk populations could help establish causality and define optimal dosing regimens. Explore Mechanistic Pathways: Further research is warranted on the molecular and neurobiological mechanisms through which vitamin D influences mood regulation, including its effects on neurotransmitter synthesis, neuroprotection, and inflammatory mediators. Advanced neuroimaging and biochemical studies could shed light on how vitamin D interacts with brain function during the critical postpartum period.

#### 4.11.3. Visual Summary of Challenges and Future Directions

Table 3 outlines the key challenges in the current body of literature and proposes future research directions.

## 5. Discussion

There is a notable association between low serum vitamin D levels and increased depressive symptoms during pregnancy and postpartum. However, evidence quality varies, and causality remains uncertain. Biological pathways, including immune modulation and hormone regulation, support a potential link, but more high quality, longitudinal studies are necessary to clarify this relationship [4]. Key insights from multiple clinical studies indicate that women with lower serum vitamin D levels, along with other nutritional deficiencies such as insufficient riboflavin, are at an increased risk of developing depressive symptoms in the postpartum period. The biological plausibility involves vitamin D’s role in brain development, neurotransmitter function, and immune regulation. While some studies show a clear association, others have inconsistent results, possibly due to differing methodologies and the timing of vitamin D assessment [5]. Most studies report an inverse relationship between vitamin D levels and depression severity, though the results vary. Some trials suggest supplementation can improve mood, but the evidence base is limited by small sample sizes and confounding factors [11]. Biological pathways enhance the credibility of a causal connection, making vitamin D supplementation a potentially low-cost intervention [4]. Potential benefits of supplementation exist but require confirmation via larger randomized trials [3]. Findings support a significant link between low vitamin D levels and PPD, reinforced by biological mechanisms like immune modulation and neuroplasticity [2]. Most studies reviewed report significant associations between low serum vitamin D levels and higher PPD severity, though some studies show no link, highlighting inconsistencies that are possibly due to methodological differences [3]. Some studies suggest supplementation reduces depressive symptoms, but variations in timing, dosage, and measurement impede firm conclusions [7]. Untreated PPD affects child growth, cognitive development, and family functioning [1]. The evidence suggests that vitamin D deficiency may serve as both a biomarker and a modifiable risk factor, advocating for routine screening prenatally [22,23]. The clinical studies reviewed indicate that women with vitamin D deficiency during pregnancy are at a heightened risk of developing PPD [8]. Optimally, maintaining adequate vitamin D levels could serve as a preventive strategy, though current evidence is limited by heterogeneity and small sample sizes [18,22]. The heterogeneity of vitamin D levels and their association with postpartum depression across populations underscores the limitations of one-size-fits-all approaches. Precision medicine approaches that integrate genetic markers, comprehensive nutritional profiles, and environmental exposures are essential for identifying women at the highest risk of PPD due to vitamin D insufficiency [2,3,5].

Individualized supplementation regimens based on precise vitamin D status and metabolic considerations have the potential to improve clinical outcomes while minimizing the risk of inefficacious or excessive dosing. Incorporating such personalized strategies aligns with growing evidence and evolving paradigms in maternal mental health care [4,14,18,22].

In addition to biochemical and genetic biomarkers, recent advances in digital mental health underscore the potential of artificial intelligence (AI) in improving the early detection and intervention of postpartum depression. AI-driven tools such as chatbots, mobile-based monitoring systems, and predictive models can analyze behavior, mood, and biological signals to support screening and personalized care. While not a replacement for clinical treatment, AI-based psychological interventions may complement traditional approaches, especially in low-resource settings or for populations with limited access to mental health professionals [24,25,26].

## 6. Conclusions

Evidence from numerous clinical studies suggests that women with low serum vitamin D levels—often combined with other deficiencies like riboflavin—face a higher risk of developing postpartum depressive symptoms. While systematic reviews report significant associations (55–71%), methodological differences, cultural factors, and confounders complicate causal interpretation. Vitamin D deficiency is widespread, especially among immigrant and postpartum populations. Clinical and biological data support a link between low levels of vitamin D and increased depressive symptoms, potentially through disrupted neurotransmitter function, weakened neuroprotection, and elevated inflammation. However, inconsistent diagnostic criteria and measurement variability call for standardized research approaches.

Given its modifiable nature, vitamin D deficiency represents a promising target for public health strategies. Screening and individualized supplementation could reduce postpartum depression risk, enhance maternal mental health, and promote healthy child development.

Future research should adopt unified protocols, focus on interventional designs, and consider the multifactorial origins of postpartum depression. Incorporating genetic and biomarker data can further refine interventions, aligning with personalized medicine principles.

Ultimately, proactive vitamin D monitoring and tailored supplementation during pregnancy offer a meaningful opportunity to reduce postpartum depression and improve the overall well-being of both the mother and child.

## Figures and Tables

**Table 1:** Summary of epidemiological studies on vitamin D deficiency and postpartum depression.

Comparative Epidemiological Findings				
Study Population	Sample Size	Vitamin D Assessment Method	Deficiency Threshold	Prevalence/Key Findings
Taiwanese Women		Plasma riboflavin and other micronutrients measured	Riboflavin notably lower in PPD group (13.9% lower)	8.4% prevalence of PPD; traditional confinement associated with higher PPD rates [18]
Iranian Women		ELISA for 25(OH)D measurement	<20 ng/mL considered deficient	Women with PPD had significantly lower vitamin D levels (16.89 vs. 21.28 ng/mL); OR: 3.3 for vitamin D < 20 ng/mL [4]
Immigrant Populations in Europe	Varied	Serum 25(OH)D concentrations via multiple assays	N/A	Lower vitamin D concentrations compared to indigenous populations; differences attributed to skin pigmentation and lifestyle [17]

**Table 2:** Comparative analysis of clinical studies on vitamin D deficiency and postpartum depression.

Study Region	Key Nutritional Findings	Depression Assessment Tool	Main Outcome	Study Limitations
Taiwan	Lower plasma riboflavin; deficient vitamin D levels	Edinburgh Post-natal Depression Scale (EPDS)	8.4% prevalence of PPD; traditional confinement linked with higher depressive symptoms	Limited by cultural confinement practices and potential confounders in nutritional status [18]
Iran	Significantly lower vitamin D levels in depressed women, with over 53% below 20 ng/mL	Beck Depression Scale	Women with vitamin D <20 ng/mL were 3.3 times more likely to develop PPD	Small sample size and potential selection bias; reliance on ELISA assay for vitamin D [5]
Europe	Immigrant populations showing lower serum 25(OH)D compared to indigenous Europeans	Various assessment tools including EPDS	Poor vitamin D status linked to higher risk of depressive symptoms	Heterogeneity in populations, sunlight exposure, and dietary intake [17]

**Table 3:** Comparison of Current Research Limitations and Proposed Future Directions.

Current Challenges	Proposed Future Directions
Heterogeneity in diagnostic criteria and study design	Standardize vitamin D deficiency thresholds and assessment tools [11]
Confounding nutritional and lifestyle variables	Incorporate comprehensive nutritional profiles and multivariate adjustment [18]
Variability in vitamin D measurement methodologies	Employ standardized and sensitive assays (e.g., LC-MS/MS) [19]
Cultural and environmental influences limiting generalizability	Conduct multi-regional, longitudinal, and culturally sensitive studies [18]

## Data Availability

The data that support the findings of this study are available from the corresponding author upon reasonable request.

## Supplementary Materials

The following supporting information can be downloaded at: https://www.mdpi.com/article/10.3390/jpm15070290/s1, Supplementary Table S1. PRISMA 2020 checklist.

## Abbreviations

The following abbreviations are used in this manuscript:

Full Term	Abbreviation
Vitamin D deficiency	VDD
Postpartum depression	PDD
Serum 25-hydroxyvitamin D	25(OH)D
Biological mechanisms	BM
