# Supplementary material for: Relationship Between Vitamin D Deficiency and Postpartum Depression
Source: J Pers Med. 2025 Jul 4;15(7):290. doi: 10.3390/jpm15070290 (PMC12298951; doi:10.3390/jpm15070290)
Supplement: Supplementary file 1 [file jpm-15-00290-s001.zip › jpm-3686966-supplementary.pdf]

Supplementary Table 1. PRISMA 2020 checklist.

PRISMA Item (No.)	Addressed	Page/s	Description and Evidence
Title (Item 1)—Identify report as review	Yes	1	The title explicitly identifies the article as a "Narrative Review," satisfying PRISMA's requirement to label the report as a review.
Abstract (Item 2)—PRISMA for Abstracts	Partially	1	A structured abstract is provided (with Background, Methods, Results, and Conclusions), but it does not fully adhere to the PRISMA 2020 abstract checklist.
Introduction (Item 3)—Rationale	Yes	2–3	The Introduction clearly explains the rationale for the review (the link between vitamin D and postpartum depression).
Introduction (Item 4)—Objectives	Yes	3	The objectives of the review are explicitly stated (to synthesize findings on vitamin D and postpartum depression).
Methods (Item 5)—Eligibility criteria	Yes	4–5	Inclusion/exclusion criteria are defined. The authors include peer-reviewed studies of perinatal populations and exclude non-English articles, studies outside the perinatal period, and commentaries/editorials.
Methods (Item 6)—Information sources	Yes	4	The information sources are described: a comprehensive search of PubMed, Scopus, and Web of Science was conducted, including the date ranges of interest.
Methods (Item 7)—Search strategy	Yes	5–6	The full search strategy is detailed: combinations of MeSH terms and keywords (e.g., "vitamin D," and "postpartum depression") with Boolean operators are provided.
Methods (Item 8)—Selection process	Yes	6	The selection process is described narratively. Two independent reviewers screened titles/abstracts and applied the eligibility criteria. The authors report screening 92 records (26 from PubMed and additional from Scopus/Web of

			Science) and including 26 studies. (No automated screening tools)
Methods (Item 9)—Data collection process	Partially	7	The data extraction process is described but not fully detailed. Data were extracted manually by reviewers (dual independent extraction and cross-verification are mentioned), without automation.
Methods (Item 10)—Data items	Yes	7	All key data items are identified: outcomes and variables (e.g., serum 25(OH)D levels, depression measures, population characteristics) are specified and recorded in tables.
Methods (Item 11)—Study risk of bias	No		The authors did not assess risk of bias in individual studies. No formal quality appraisal tool was applied (as noted, "No—Not assessed" because this is a narrative review).
Methods (Item 12)—Effect measures	No		No effect measures (e.g., risk ratios) are reported, as no meta-analysis was performed (not applicable).
Methods (Item 13)—Synthesis methods	Yes	7–8	A thematic synthesis approach is described: findings are organized by themes rather than pooled quantitatively. The narrative synthesis methodology is explicitly stated.
Methods (Item 14)—Reporting bias assessment	No		The review does not report any assessment of reporting (publication) bias. No methods (e.g., funnel plots) were used.
Methods (Item 15)—Certainty assessment	No		The certainty (or confidence) of the body of evidence was not evaluated.
Results (Item 16)—Study selection flow diagram	No		No PRISMA flow diagram is provided. The authors instead describe the selection process in text (92 records screened and 26 included).
Results (Item 17)—Study characteristics	Yes	8–9	Characteristics of included studies (design, sample, and measures) are summarized in tables (e.g., Table 1). This fulfills the requirement to present study features.
Results (Item 18)—Risk of bias in studies	No		The review did not present risk-of-bias assessments for the included studies.
Results (Item 19)—Results of individual studies	Yes	8–11	Findings from each study are reported in the text and table form, allowing readers to see individual

			study results (though effect sizes are not pooled).
Results (Item 20)—Results of syntheses	Yes	11–13	The results are synthesized qualitatively: themes and patterns across studies are described (a narrative synthesis of the evidence).
Results (Item 21)—Reporting biases	No		Reporting bias (e.g., publication bias) is not evaluated in this review.
Results (Item 22)—Certainty of evidence	No		Certainty or confidence in the cumulative evidence is not assessed.
Discussion (Item 23)—Discussion of results	Yes	14	The Discussion integrates the review findings with existing knowledge and theory, addressing how the results support a link between vitamin D and PPD.
Discussion (Item 24)—Limitations of evidence	Yes	15	Limitations of the evidence (e.g., heterogeneity and methodological variability) are explicitly discussed.
Discussion (Item 25)—Limitations of review process	Yes	15–16	The authors acknowledge limitations of their review process (e.g., lack of bias assessment tools).
Discussion (Item 26)—Implications	Yes	16	Implications for practice (e.g., screening for deficiency) and future research are clearly articulated.
Other (Item 27)—Registration and protocol	No	16	No protocol was registered or published (the authors note "No—Not registered").
Other (Item 28)—Support/funding	Yes	16	The funding statement declares no external funding for the work.
Other (Item 29)—Competing interests	Yes	17	The authors declare no competing interests.
Other (Item 30)—Availability of data/code	Yes	17	The data supporting the study are available from the authors on request.
